# Functionalization of Electrospun Nanofibers and Fiber Alignment Enhance Neural Stem Cell Proliferation and Neuronal Differentiation

**DOI:** 10.3389/fbioe.2020.580135

**Published:** 2020-10-26

**Authors:** Miriam C. Amores de Sousa, Carlos A. V. Rodrigues, Inês A. F. Ferreira, Maria Margarida Diogo, Robert J. Linhardt, Joaquim M. S. Cabral, Frederico Castelo Ferreira

**Affiliations:** ^1^Institute for Bioengineering and Biosciences, Department of Bioengineering, Instituto Superior Técnico, Universidade de Lisboa, Lisbon, Portugal; ^2^Center for Biotechnology and Interdisciplinary Studies, Department of Chemistry and Chemical Biology, Rensselaer Polytechnic Institute, Troy, NY, United States

**Keywords:** PCL, functionalization, nanofibers, electrospinning, neural stem cells, laminin, GRGDSP, alignment

## Abstract

Neural stem cells (NSCs) have the potential to generate the cells of the nervous system and, when cultured on nanofiber scaffolds, constitute a promising approach for neural tissue engineering. In this work, the impact of combining nanofiber alignment with functionalization of the electrospun poly-ε-caprolactone (PCL) nanofibers with biological adhesion motifs on the culture of an NSC line (CGR8-NS) is evaluated. A five-rank scale for fiber density was introduced, and a 4.5 level, corresponding to 70–80% fiber density, was selected for NSC *in vitro* culture. Aligned nanofibers directed NSC distribution and, especially in the presence of laminin (PCL-LN) and the RGD-containing peptide GRGDSP (PCL-RGD), promoted higher cell elongation, quantified by the eccentricity and axis ratio. *In situ* differentiation resulted in relatively higher percentage of cells expressing Tuj1 in PCL-LN, as well as significantly longer neurite development (41.1 ± 1.0 μm) than PCL-RGD (32.0 ± 1.0 μm), pristine PCL (25.1 ± 1.2 μm), or PCL-RGD randomly oriented fibers (26.5 ± 1.4 μm), suggesting that the presence of LN enhances neuronal differentiation. This study demonstrates that aligned nanofibers, functionalized with RGD, perform as well as PCL-LN fibers in terms of cell adhesion and proliferation. The presence of the full LN protein improves neuronal differentiation outcomes, which may be important for the use of this system in tissue engineering applications.

## Introduction

Neural stem cells (NSCs) are self-renewing multipotent cells with the capacity to differentiate into neurons and glial cells (astrocytes and oligodendrocytes) (Pollard et al., 2006b; Conti and Cattaneo, 2010). The use of NSCs combined with engineered biomaterials has the potential to provide new therapeutic routes, including the regeneration of the central nervous system (CNS) when impaired by neurodegenerative diseases, such as Alzheimer or Parkinson diseases, or traumatic injuries such as spinal cord injury (Grochowski et al., 2018; Pereira et al., 2019). Moreover, NSCs can be used to generate *in vitro* disease models (Jakel et al., 2004; Zhao and Moore, 2018), which may be important tools to provide new insights into disease mechanisms, as well as to discover and test new drugs (Gorba and Conti, 2013).

Neural stem cell-based therapeutic strategies may involve the stimulation of endogenous stem cells or on the transplantation of exogenous stem cells previously expanded *in vitro*. The use of biomaterial scaffolds provides an adequate surface for cell adhesion, enabling efficient cell proliferation, differentiation, and organization into a mature and functional engineered tissue (Kim et al., 2012). The role of the biomaterial scaffold also provides appropriate mechanical and physicochemical properties to the new tissue, as well as a geometry that contributes to cell organization (e.g., cell alignment) (Schaub et al., 2016).

The NSC niche is a complex structure, with a specific extracellular matrix (ECM) composition, able to support NSC maintenance and differentiation *in vivo*. The components of the ECM interact with cells through transmembrane proteins, called integrins, which trigger intracellular signaling pathways, influencing cell function and cell fate (Flanagan et al., 2006; Wang et al., 2011). Culture substrates processed from natural and synthetic materials have been developed to mimic the role of the native ECM on the support of NSCs (Ciardelli et al., 2005; Kim and Park, 2006; Hiraoka et al., 2009; Ananthanarayanan et al., 2010; Hackett et al., 2010; Cooper et al., 2011; Nakaji-Hirabayashi et al., 2012). Among the latter, polyesters such as poly-ε-caprolactone (PCL), polylactic acid, polyglycolic acid, and their copolymers are of particular interest in regenerative medicine as scaffolds. Such polymers are biocompatible and biodegradable and have been approved by the regulatory entities for medical applications (Schaub et al., 2016). However, the use of these synthetic biomaterials as substrates for cell culture often requires functionalization with specific biological motifs, namely, ECM proteins such as laminin” (LN), which is a protein found in the basement membrane and described to support NSC adhesion, migration, and differentiation (Hall et al., 2008; Koh et al., 2008; Hiraoka et al., 2009; Klinkhammer et al., 2010). The use of synthetic peptides, with cell adhesion motifs recognizable by integrins, for biomaterial functionalization has also been described and circumvents the issues raised by use of proteins of animal origin (batch to batch variability, pathogen, and immunogenic contamination), also being more cost-effective (Hersel et al., 2003; Hall et al., 2008; Ananthanarayanan et al., 2010). The arginine–glycine–aspartate peptide, or RGD, is a small amino acid sequence, which is conserved in nature and is present in many ECM proteins, including fibronectin and LN (Hersel et al., 2003; Causa et al., 2010; Gloria et al., 2012).

Nanofiber matrices, which can be produced by electrospinning, are promising structural substrates for NSC culture because this configuration can provide specific geometries at the cell scale, capable of reproducing the native tissue architecture (Xue et al., 2020). The nanofiber mesh affords a high surface-to-volume ratio while providing high porosity and permeability, permitting suitable diffusion of nutrients, metabolites, and gases. The substrate morphology and the presence of specific biochemical cues (adhesion molecules and growth factors) are critical to control cellular fate *in vitro*, impacting, for instance, cellular adhesion and morphology (elongation, spreading) (Beachley and Wen, 2009; Ghasemi-Mobarakeh et al., 2010; Hackett et al., 2010; Gloria et al., 2012). Previous studies have shown the importance of using electrospun fibers to culture NSCs from different sources, using fiber geometry to promote tissue organization. Aligned PLLA nanofibers promoted NSC alignment and neurite extension according to fiber alignment direction (Yang et al., 2005). Neural precursors, derived from human embryonic stem cells (ESCs), were cultured on aligned LN-coated PCL fibers, showing a more accentuated polar morphology, increased neuronal differentiation, and neurite extension along the fiber direction (Mahairaki et al., 2011). LN-coated aligned PCL nanofibers were used to culture adult rat NSCs, promoting accentuated cellular alignment, neurite extension along the fiber alignment axis, and also a higher number of differentiated Tuj1^+^ cells (Lim et al., 2010). NSCs with more polarized and elongated morphology were also obtained in LN-coated aligned polystyrene nanofibers, together with high neuronal lineage differentiation (Bakhru et al., 2011). Different peptides containing biological motifs have been incorporated into synthetic materials to improve cell adhesion to the nanofibers, as well as differentiation outcomes, with higher reproducibility and manufacture standardization. Examples of these studies include the culture of mouse ESCs in aligned and functionalized fibers with YIGSR, a peptide derived from LN, which leads to increased expression of neuronal markers (Tuj1) and neurite extensions when compared to random and non-functionalized fibers (Smith Callahan et al., 2013). Aligned fibers, functionalized with epidermal growth factor (EGF) (Lam et al., 2010), were used to culture human ESC-derived NSCs, leading also to higher expression of glial and neuronal markers and axon extension. Cortical NSCs had higher proliferation and preferentially differentiate into oligodendrocytes and neurons in both randomly oriented and aligned brain-derived neurotrophic factor (BDNF)–functionalized PCL nanofibers (Horne et al., 2009). Glial cell-derived neurotrophic factor–functionalized PCL fibrous scaffolds (Wang T.Y. et al., 2012) were also successfully used to culture neural stem/progenitor cells with increased cell viability, proliferation and neurite outgrowth, upon transplantation. PCL nanofibers were also functionalized with the GYIGSR or RGD peptides and used to study the impact of these peptides and fiber morphology on mouse ESC neural differentiation (Silantyeva et al., 2018; Philip et al., 2019). PCL fibers functionalized with GYIGSR accelerated neural differentiation, whereas the use of RGD nanofibers promoted enhanced GFAP expression. Guidance of neurites parallel to the fiber direction was observed in both cases, when aligned fibers were used.

In this work, a detailed comparative study was performed, to understand the importance of combining fiber organization and selected biological motifs on NSC proliferation, differentiation, and morphology. PCL was selected because of its slow biodegradability (in the range of 1–2 years), which ensures support of cells during slow tissue regeneration *in vivo*. PCL nanofibers with different morphologies, random and aligned, were functionalized with adhesion factors that promote NSC elongation, namely, LN, a complete protein widely used to promote cell adhesion and neural differentiation, or GRGDSP, one of the most active RGD-containing peptides for recognition by cell adhesion molecules (Hautanen et al., 1989; Hersel et al., 2003). The functionalized nanofibers were used to study the impact of the selected ECM motif over NSC proliferation, differentiation, and cellular morphology. The CGR8-NS cell line, derived from the mouse embryonic cell line CGR8, was selected as NSC model. This robust cellular model can be stably expanded *in vitro* and maintain neuronal and glial differentiation even after long-term passaging (Conti et al., 2005; Pollard et al., 2006a). Moreover, these cells proliferate adherent to physical supports as a cell monolayer (Rodrigues et al., 2010, 2011), which is of particular interest to easily assess the effect of fibers with different morphologies on cell populations including their orientation and shape. The results obtained show, in a comparative manner, the impact of the different biological motifs in combination with fiber geometry, on cell attachment, proliferation, and differentiation, as well as on cell alignment and morphology. This study demonstrates that despite small peptide motifs (GRDGSP) can provide equivalent results to LN in terms of cell adhesion and proliferation, the use of the full LN protein has advantages in terms of neuronal differentiation. The current work also presents for the first time a five-rank scale for fiber density, which allows us to standardize the nanofiber scaffolds prepared and improves the reproducibility of the experiments.

## Materials and Methods

### PCL Nanofiber Preparation by Electrospinning

**FIGURE 1:**
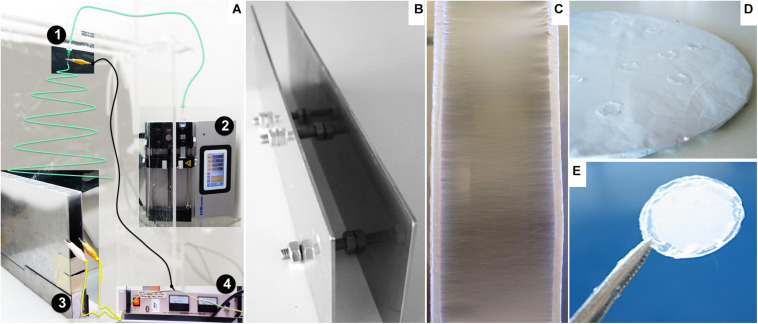
Nanofiber preparation and assembly: **(A)** Adapted view of the electrospinner apparatus: 1: needle, 2: syringe pump, 3: parallel plate collector, 4: power source. The polymer solution contained in the syringe is loaded at a constant flow by the pump (2) along a tube (green line) to the needle (1) positioned above and perpendicular to the grounded collector (3). The power source equipment (4) provides the electric potential to charge the polymer solution, wired to the stainless steel tip of the needle by the black cable, while permitting grounding the collector connected by the yellow wires. **(B)** Parallel plates used as a collector for aligned nanofibers. **(C)** Detailed view of deposited aligned nanofibers oriented perpendicular to the edges of the plates. **(D)** Round flat collector covered with a random fiber mesh. **(E)** Nanofiber sample fixed on a glass slide.

Aligned and randomly distributed PCL nanofibers were prepared using an electrospinning apparatus, as described elsewhere (Canadas et al., 2014). The equipment setup (Figure 1A), assembled inside a fume hood, was composed of a high-voltage power supply (Model PS/EL40P0, Series EL 1; Glassman High Voltage Inc., High Bridge, NJ, United States), a syringe pump (Model KDS Legato 210; KDS Scientific, Holliston, MA, United States), and a tube connecting a syringe (Henke Sass Wolf, Germany) to a needle (Needle Valve Dispense Tip Kit; EFD International Inc., United Kingdom) with an inner diameter of 0.84 mm. Two types of collectors were used: two parallel steel plates with a 2-cm gap collector (Figures 1B,C) and a flat copper plate (Figure 1D), positioned below and perpendicular to the needle, as reported in the literature (Li et al., 2003; Teo and Ramakrishna, 2006; Beachley and Wen, 2009). Operational parameters applied on the electrospinning process were previously optimized in-house (data not shown). The nanofibers were prepared with 6% wt/wt solution of PCL (70,000–90,000 MW; Sigma–Aldrich, St. Louis, MO, United States) in 1,1,1,3,3,3-hexafluoro-2-propanol (HFP; Sigma–Aldrich) at a flow rate of 1 mL h^–1^, with an applied electrical potential and working distance (tip of the needle to the nanofiber deposition target) of 26 kV and 20 cm or 30 kV and 35 cm to produce aligned or randomly distributed nanofibers.

The fibers were carefully collected from the supports and fixed onto glass coverslips (13-mm diameter; VWR, Radnor, PA, United States) with medical-grade biocompatible silicone glue (Silastic Medical Adhesive Silicone type A; Dow Corning, Midland, MI, United States), ensuring that the mesh maintained structure integrity throughout the experiments (Figure 1E). The ranges for humidity and temperature working conditions were 30–40% and 22–25°C, respectively.

The fiber alignment was estimated by measuring the angle of each fiber relative to a horizontal reference line. The angle values were normalized and represented in a histogram within a range of −90° and +90°.

We used both optical microscopy images and higher-resolution scanning electron microscopy (SEM) images of different fiber series to establish the “five-scale fiber-density ranking.” The images were analyzed using ImageJ, being converted in bimodal images (with only black and white pixels) to estimate the ratio between empty space and fibers (Wang et al., 2010).

### Functionalization of the PCL Nanofibers

#### Aminolysis Reaction

The nanofibers were washed with a 50% vol/vol solution of ethanol (Thermo Fisher Scientific, Waltham, MA, United States) in water for 1 h and rinsed with deionized water under gentle agitation, at room temperature. The aminolysis reaction took place immersing the samples in 10% wt/vol 1,6-hexanediamine (HDA, Fluka, Germany) in isopropanol (Thermo Fisher Scientific) for 40 min at 37°C, as described elsewhere (Zhu et al., 2002). After aminolysis, the nanofibers were rinsed with deionized water five times.

#### Protein Immobilization

Solutions of Laminin (LN, 20 μg mL^–1^; Sigma–Aldrich) and of the peptide glycine–arginine–glycine-aspartic acid–serine–proline (GRGDSP, 50 μg mL^–1^; Sigma–Aldrich) were prepared in phosphate-buffered saline (PBS; Life Technologies, Waltham, MA, United States). Protein or peptide crosslinking to the amine group, previously introduced in the PCL fibers by aminolysis, was performed by reaction over 24 h in glutaraldehyde atmosphere (Migneault et al., 2004), using a solution of 2.5% vol/vol glutaraldehyde (GA; Sigma–Aldrich). Afterward, the samples were washed with PBS five times and immersed in a solution of glycine (100 mg mL^–1^ in PBS; Sigma–Aldrich) for 1 h at room temperature, to quench free aldehyde groups. Finally, the samples were washed again five times with PBS at room temperature.

#### Quantification of Immobilized Protein

The quantity of protein or peptide attached to the scaffold surface was estimated using the colorimetric ninhydrin assay, which quantifies the total amine groups (Zhu et al., 2002; Friedman, 2004). This method is based on the reaction of the amine groups with ninhydrin, resulting in the formation of a blue compound measurable by absorbance spectroscopy. Nanofiber samples of equivalent dimensions (nanofiber mesh covering approximately 0.8 cm^2^ of surface area) were removed from the glass slides, immersed in 0.5 mL of 1.0 mol L^–1^ ninhydrin (Merck, Germany) in ethanol for 1 min at room temperature and heated at 80°C for 20 min, until complete ethanol evaporation. To dissolve the PCL mesh sample, 0.5 mL of 1,4-dioxane (Thermo Fisher Scientific) was added, followed by 0.5 mL of isopropanol, to stabilize the blue compound formed. Pristine PCL fibers, without any chemical treatment, were used as control for any non-specific residual chromophore response, and PCL fibers submitted to aminolysis, but without protein functionalization, were used as an additional control. The absorbance of the reaction product was measured at 538 nm using a microplate reader (Infinite M200 Pro, Tecan, Switzerland). A reference calibration curve was obtained measuring the absorbance of ninhydrin–NH_2_ product as a function of graded concentrations of HDA in 1:1 vol/vol of 1,4-dioxane/isopropanol solutions (Supplementary Figure 1D).

### NSC Culture

The cell model used was the NSC line CGR8-NS, derived from the mouse ESC line CGR8 (Conti et al., 2005) and provided by the laboratory of Professor Austin Smith (Welcome Trust Centre for Stem Cell Research, Cambridge, United Kingdom).

#### CGR8-NS Culture in Standard Polystyrene Surface

The NSC culture was performed as previously described (Conti et al., 2005; Rodrigues et al., 2010). Cryopreserved CGR8-NS cells, upon thawing, were expanded on uncoated tissue culture T-flasks or 24-well plates (Falcon; BD Biosciences, San Jose, CA, United States), in serum-free NSC expansion medium composed of Dulbecco modified eagle medium (DMEM)/F12 + Glutamax (Thermo Fisher Scientific) supplemented with 1% vol/vol N2 (Thermo Fisher Scientific), 20 ng mL^–1^ of both FGF-2 and EGF (PeproTech, Rocky Hill, NJ, United States), 0.1% vol/vol B27 (Life Technologies), 1% vol/vol penicillin–streptomycin (10,000 U mL^–1^, Thermo Fisher Scientific), 1.6 g L^–1^ glucose (Sigma–Aldrich), and 20 mg L^–1^ insulin (Sigma–Aldrich). The cells were cultured at 37°C under 5% CO_2_ humidified atmosphere and maintained at passages between 45 and 54. Each passage was performed at 80–90% confluence. Cells were harvested using Accutase (Sigma–Aldrich), and cell viability was evaluated using the trypan blue (Thermo Fisher Scientific) exclusion method (Strober, 2001) by direct counting of viable cells in a hemacytometer, under an optical microscope (Olympus, Germany). Cell viability remained greater than 90%.

#### CGR8-NS Culture on the PCL Nanofibers

The nanofibers were placed in sterile 24-well ultralow attachment cell culture plates (Corning, NY, United States) and sterilized with antibiotic–antimycotic (Thermo Fisher Scientific) solution overnight. After sterilization, the nanofibers were washed with sterile PBS and rinsed with culture medium before cell seeding. A suspension of 100 μL with 2.0 × 10^5^ CGR8-NS cells in fresh supplemented medium was deposited carefully on top of each nanofiber and incubated for 1 to 2 h to promote initial cell deposition and adhesion to the material.

The seeding density was defined considering a previously optimized value of 1.0 × 10^4^ cells cm^–2^ (Rodrigues et al., 2010) and also the surface available for cells to adhere to the nanofibers as being at least three times higher than the flat surface of the well of the tissue culture plate. Reported in *Functionalization of the Nanofiber Surface*, a ratio 6:1 of fibers per flat surface was estimated, so the cell density was increased accordingly. After cell adhesion to the nanofibers, culture medium was added up to final volume of 0.5 mL. CGR8-NS cells (2.5 × 10^4^ cells cm^–2^) were also cultured in standard uncoated 24-well tissue culture plates as a control (Supplementary Figure 2).

#### Evaluation of Cell Growth

Viability and estimation of CGR8-NS cell number were monitored indirectly over time using Alamar Blue (Thermo Fisher Scientific) according to the manufacturer instructions and through a calibration curve (Supplementary Figure 3) relating the fluorescence intensity with the number of CGR8-NS cells, counted using a hemocytometer. Fluorescence was measured using a microplate reader at excitation and emission wavelengths of 560 and 590 nm, respectively.

The cell growth rate (μ) was determined using an “Ln X versus time” plot, as the slope of a linear regression line, according to the following (1):

(1) $$\text{Ln}\,\left(\text{X}\right)=\mathrm{Ln}\,\left({\text{X}}_{0}\right)+\mu \,t$$

where *X* is the cell number and *X*_0_ the initial cell number. The doubling time was obtained by (2):

(2) t1⁢/⁢2=Ln⁢ 2⁢/⁢μ⁢

#### CGR8-NS Differentiation

After 11 days of NSC expansion on the nanofibers, a neuronal differentiation protocol was adapted (Pollard et al., 2006a) and performed *in situ*. At day 1 of differentiation, NSC medium (as described in *CGR8-NS Culture in Standard Polystyrene Surface*) was refreshed. On the next day, medium was replaced, this time without EGF, and with 5 ng ⋅ mL^–1^ of FGF-2 and 2% vol/vol B27. Half the medium was replaced after 4 days. At day 9, culture medium was replaced by a 1:1 mixture of DMEM/F12 and neurobasal medium (1×) (Thermo Fisher Scientific) without EGF or FGF-2 and with 2% vol/vol B27. Half the medium was replaced after 4 days, and culture was maintained until day 15 of differentiation. Supplementary Figure 4 summarizes the applied protocol.

### Cell Staining and Immunocytochemistry

The spatial distribution and morphology of CGR8-NS cells on the nanofibers were qualitatively assessed by labeling the nuclei with 4’,6-diamino-2-phenylindole (DAPI; Sigma–Aldrich) and F-actin filaments of the cytoskeleton with the fluorescent dye rhodamine phalloidin (Thermo Fisher Scientific). CGR8-NS cells were fixed with 4% paraformaldehyde (PFA; Sigma–Aldrich) for 10 min at room temperature, washed once with PBS, and permeabilized with 0.1% vol/vol Triton X-100 (Sigma–Aldrich) and 5% vol/vol normal goat serum (NGS; Sigma–Aldrich) in PBS for 15 min. The cells were stained with 300 μL of rhodamine phalloidin probe (0.2 μg mL^–1^ in PBS) for 45 min at room temperature. After washing once with PBS, the cells were incubated with 300 μL of DAPI (1.5 μg mL^–1^ in PBS) for 5 min. Finally, the cells were washed two times in PBS. Cells were visualized under a fluorescence optical microscope (DMI 3000B; Leica, Germany). Digital images were taken with a digital camera (DXM 1200F; Nikon, Japan).

Immunophenotype analysis was performed for Sox2, Nestin, Tuj1, and GFAP antibodies. The cells were fixed in 4% PFA for 10 min at room temperature, washed once with PBS, and permeabilized with 0.1% vol/vol Triton X-100 and 10% vol/vol NGS in PBS for 1 h at room temperature. Primary antibodies were incubated overnight at 4°C in 0.1% vol/vol Triton X-100 and 5% vol/vol NGS in PBS. The following primary antibodies were used: anti-Sox2 (1:100, R&D Systems, MN, United States), anti-Nestin mouse monoclonal antibody (1:200, Millipore, Germany), anti–βIII-tubulin (Tuj1,1:2000, Covance, Princeton, NJ, United States), and anti–glial fibrillary acidic protein GFAP (1:100, GFAP; Millipore). After primary antibody incubation, the cells were washed once with PBS and incubated with the proper secondary antibody conjugated with Alexa Fluor 546 (1:500; Life Technologies) for 1 h at room temperature, protected from light. Next, the cells were washed with PBS and nuclei stained with DAPI (1.5 μg mL^–1^ in PBS) for 5 min at room temperature. Finally, the cells were washed two times in PBS and visualized under a fluorescence optical microscope.

### Scanning Electron Microscopy

The nanofibers and cell morphology were examined by SEM. Scaffold samples containing cells were fixed with 4% PFA for 15 min, washed once with PBS, and dried by immersion in graded concentrations of ethanol solutions in water (25, 50, 75, and 100% vol/vol). The samples were kept in an aseptic environment until complete drying. Prior to SEM visualization, the samples were coated with a 45-nm gold/palladium layer by a sputter coater (model E5100, ex-Polaron; Quorum Technologies, ON, Canada) and observed under a conventional SEM (model S2400; Hitachi, Japan) with an electron beam with 20 kV of accelerating voltage. SEM images were analyzed with ImageJ (National Institutes of Health, United States) to estimate both orientation and fiber diameter profiles. At least 50 samples were individually measured for each condition.

### Cell Shape Analysis

The eccentricity is a parameter that can be used to describe the cellular shape (Xie et al., 2009a,b). The NSC bipolar shape obtained from SEM images was fitted using ImageJ to an elliptical geometric form, to determine the major and minor axis. The eccentricity was calculated with Equation (1),

(3) $$\mathrm{Eccentricity}=\frac{\sqrt{\left({\text{a}}^{2}-b^{2}\right)}}{a}$$

where *a* and *b* are the semimajor and semiminor axis of the ellipse, respectively. Eccentricity values vary between 0 (which corresponds to a circle) and 1 (in this limit closest to a line segment).

Additionally, the elongation of the elliptical form measured was evaluated by the ratio between the minor and the major axis that, inversely, when equal to 1 corresponds to a perfect circle and when closest to 0 describes a shape approaching a line segment.

### Statistical Analysis

The results are expressed as mean ± standard error of the mean. Statistical analysis was performed with ordinary one-way analysis of variance for multiple group comparison tests. Statistical comparison between two groups was performed with an unpaired *t* test. Statistically significant results were considered for *p* < 0.05.

## Results

### Nanofiber Alignment, Diameter, and Density

**FIGURE 2:**
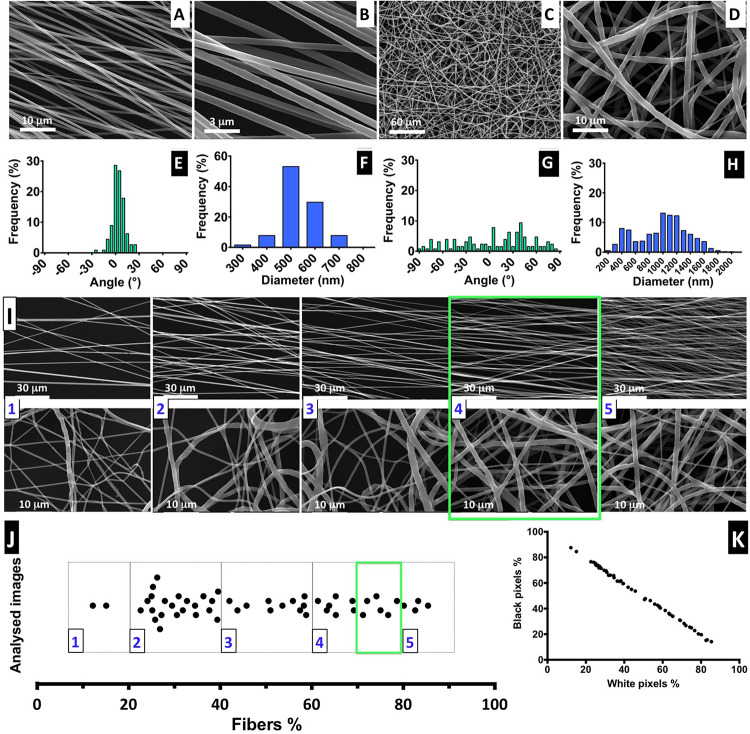
Fiber characterization: **(A–D)** SEM images of aligned and randomly distributed fibers (fiber density 70–80%). **(E–H)** Histograms of alignment profile and fiber diameter distribution. At least 100 fibers were measured in each case. **(I)** SEM images for aligned and random fibers, ordered from low- to high-density mesh; scale bars: 30 and 1 μm for the aligned and random meshes, respectively. **(J)** Distribution of analyzed images with correspondent percentage of fiber mesh, divided into five degrees of density percentage; highlighted with a green square is the interval of fiber density of the samples selected for the cell culture experiments. **(K)** Proportion of the black and white pixels that compose the bimodal images.

The electrospinning conditions were optimized for a solution of 6% PCL to produce constant, uniform, and reproducible deposition of aligned and random defect-free nanofibers with smooth surface morphology. In Figure 2, SEM images of the prepared nanofibers are shown, as well as the distribution of diameters and relative orientation angles. Figures 2A,B show images of aligned fibers at low and high magnification, respectively. Representative images of randomly organized fibers at low (Figure 2C) and high magnification (Figure 2D) are also presented. Figures 2A,D are shown at the same magnification. The estimated average diameter of the aligned nanofibers was 0.54 ± 0.08 μm, with more than 90% of the fibers oriented within a range of ±30° angle to a reference axis, evidencing a clear uniaxial disposition (Figure 2E). The random fibers obtained present a wide dispersion, with the fiber angles relative to the reference axis (Figure 2G) covering all the angle range and with approximately only 35% fibers oriented within the narrower range of ± 30°; this feature highly contrasts the tight distribution found for the aligned fibers. Also, the random fibers have more heterogeneous diameters, in a range of 0.32 to 1.55 μm (average = 0.99 ± 0.37 μm) (Figure 2H). The difference between aligned and random fiber diameters and dispersion profile can be explained by the use of different collectors (Figure 1). The two-parallel-steel-plates electrode collector promotes the formation of fibers stretched on the 2-cm gap between the two narrow edges of the plates. Such process promotes not only fiber alignment, but also more uniform and smaller fiber diameters. The flat copper plate electrode provides a wide surface for fiber deposition promoting a broader electrical field shape and dispersion of fiber formation paths, thus leading to a wider range of diameter sizes. The flat copper plate is much less effective than the parallel-plate collector on stretching the fibers upon their formation and on promoting solvent evaporation from the deposited fibers. These factors can contribute to the existence of larger diameter fibers on randomly organized fiber scaffolds.

The electrospinning setup used for producing the nanofibers is non-automatic and performed manually, but it is challenging to control the density of the deposited fiber mesh produced. Therefore, to ensure the fiber meshes used in cell culture have a similar density, we implemented a post-manufacturing step of fiber mesh sorting, in which we suggest the introduction of a “five-scale fiber-density ranking” method. Representative SEM images to illustrate each level of density are shown in Figure 2I, for both aligned and randomly distributed fiber meshes, and the respective images obtained by optical microscopy are presented in Supplementary Figure 5.

The quantification of fiber percentage (from approximately 10–90%) and the number of analyzed nanofiber SEM images, distributed by the five levels, is shown in Figure 2I. In Figure 2K, the proportion of both black and white pixels that compose the bimodal images is presented. For the NSC culture work performed in this study, the nanofiber meshes used were sorted based on optical microscopy examination to fall into 4.5 level of the “five-scale fiber-density ranking.” This level, highlighted in the green square of Figures 2I,J, corresponds to a 70–80% fiber density and was selected because the nanofiber mesh was not too dense, allowing us to visualize the cells and fibers, while avoiding cell growth on the underlying glass coverslip.

### Functionalization of the Nanofiber Surface

The next step for the preparation of the nanofibers for cell culture was to bind LN and the GRGDSP peptide on the material surface. The aligned PCL nanofiber meshes were identified as “PCL,” for non-modified pristine PCL nanofibers, “PCL-NH_2_” for aminolysed PCL nanofibers, and “PCL-LN” or “PCL-RGD” for LN and GRGDSP functionalized nanofibers, respectively. The random PCL fibers functionalized with GRGDSP were identified as PCL-RGDr. Examples of SEM images of the PCL nanofibers after aminolysis show structural integrity with no alteration on morphology, and the reaction schemes for the formation of the treated material PCL-NH_2_ are presented in Supplementary Figures 1A,B, respectively.

The overall surface area of the scaffolds fiber mesh was estimated to be 5 cm^2^ from SEM imaging covering 0.8 cm^2^ of glass slide, which corresponds to a ratio of approximately 6:1 cm^2^ of fiber surface per slide. The engrafted NH_2_ groups on the polymer surface and the immobilized LN and GRGDSP peptide were both evaluated by the ninhydrin method (Zhu et al., 2002; Kim and Park, 2006), which quantifies the equivalent free amine groups (Supplementary Figure 1). This method quantifies amines without discriminating whether peptide or protein is immobilized to the fibers surface covalently or through weak physiochemical interactions (adsorption). In this work, to minimize the contribution of adsorption, the samples were extensively washed before the ninhydrin assay. However, the chemical strategy carried out herein (i.e., ester aminolysis followed by reaction with glutaraldehyde for the functionalization of PCL nanofibers with the protein/peptide) follows the methodology reported in previous studies (Zhu et al., 2002) for immobilization of gelatin, and collagen, where PCL functionalization was demonstrated by *x*-ray photoelectron spectroscopy and contact angle measurements.

Regardless of the limitations on the ninhydrin method, the amount of NH_2_ groups per mesh surface area was estimated at a value of (7.1 ± 0.8) nmol cm^–2^ for PCL-NH_2_ samples (not quenched with glycine), assuming insertion of functional amine groups onto the nanofibers (Supplementary Figure 1C). Estimated equivalent amine densities for the PCL-LN and PCL-RGD samples, at values of (28.1 ± 0.8) and (277.2 ± 61.2) nmol cm^–2^, respectively, were higher than for PCL-NH_2_ samples. In pristine PCL fibers, a background misreading absorbance was estimated as 2.4 ± 0.5 nmol cm^–2^. By comparing the ninhydrin assay results for the initial solutions used in the crosslinking reaction and for the respective nanofiber meshes (Supplementary Figure 1C), an efficiency of nanofiber functionalization was estimated as 22.1 ± 1.5% and 83.8 ± 16.8% for PCL-LN and PCL-RGD, respectively.

### NSC Proliferation on the Nanofiber Scaffolds

**FIGURE 3:**
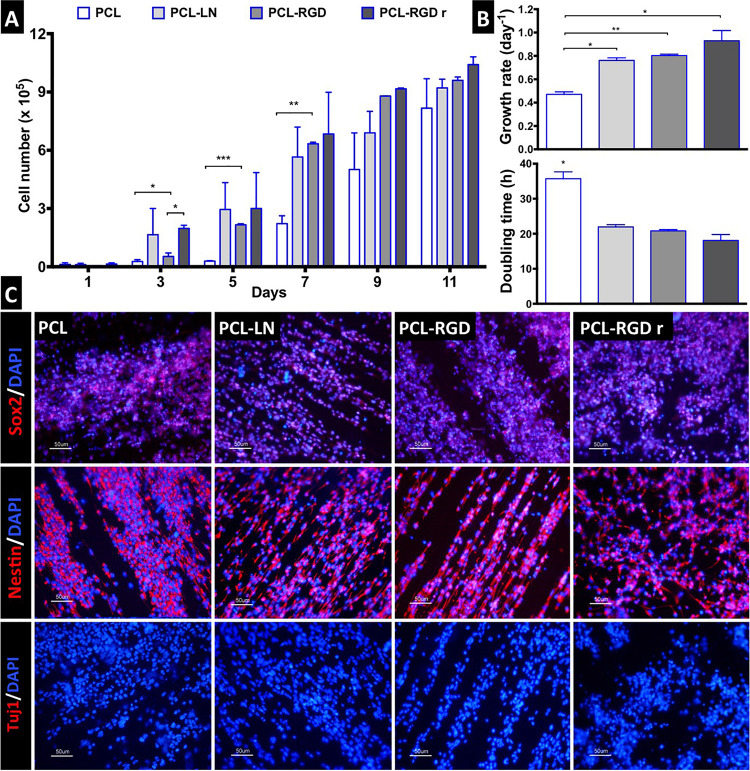
CGR8-NS growth profile on aligned PCL, PCL-LN, PCL-RGD, and random PCL-RGD nanofibers: **(A)** Cell number variation over 11 days of culture (*n* = 2); values correspond to initial cell seeding of 2.0 × 10^5^ cells per scaffold. **(B)** Growth rates (day^–1^) and doubling times (h) for each condition; error bars are standard error of the mean; *p < 0.05, **p < 0.01, ***p < 0.001. **(C)** Fluorescence microscopy images of immunocytochemistry for Nestin, Sox2, and βIII–tubulin/Tuj1. Nuclei counterstained with DAPI; scale bar = 50 μm.

The NSC proliferation profile on the nanofiber scaffolds is represented in Figure 3A. In general, the number of cells increased over time in all the conditions. Analyzing in details of the 11 days of the cell culture, an initial drop in cell number is observed in the beginning of the culture (day 1). The initial cell number (2.0 × 10^5^ cells) was achieved after only 3 days, in the case of PCL-LN and PCL-RGDr; after 5 days, for cultures in PCL-RGD aligned; and after 7 days, for pristine PCL fibers. Significantly higher cell numbers were observed on functionalized PCL-RGD fibers, relative to pristine PCL, at days 7 and 9.

However, these differences decreased and turned out fairly reduced by day 11. The scaffold area is similar for all the conditions, and when its maximum capacity for cell support is used, cell proliferation becomes reduced, indicating that cell confluence may have been reached, and therefore, differences between cell numbers are dissipated in the functionalized scaffolds by day 11.

The cell numbers at the end of the culture were (8.2 ± 1.5) × 10^5^, (9.2 ± 0.4) × 10^5^, (9.6 ± 0.2) × 10^5^, and (1.0 ± 0.4) × 10^6^ cells for PCL, PCL-LN, PCL-RGD and PCL-RGDr, respectively. The lower growth rate and higher doubling time were determined (Figure 3B) for cultures on pristine PCL (0.47 ± 0.02 day^–1^ and 35.7 ± 1.9 h). In contrast, higher cell growth rates and lower doubling times were observed in PCL-LN (0.76 ± 0.02 day^–1^; 21.9 ± 0.6 h), PCL-RGD (0.80 ± 0.01 day^–1^; 20.8 ± 0.4 h), and PCL-RGDr (0.92 ± 0.09 day^–1^; 18.1 ± 1.7 h). No major differences in terms of growth kinetics were observed between aligned and random matrices (PCL-RGD).

The cell quality after 11 days of culture on the PCL nanofiber scaffolds was evaluated by immunocytochemistry analysis for specific NSC markers (Nestin, Sox2) and for a neuronal differentiation marker (Tuj1/βIII–tubulin), as shown in Figure 3C. It was observed that Sox2 and Nestin are expressed in all the conditions, whereas βIII–tubulin expression was never detected. Moreover, the immunostaining images provide evidence of the impact of the aligned structure of the substrate on the cellular organization on the nanofibers, being clear that cellular distribution follows the nanofiber arrangement.

### Evaluation of NSC Organization and Morphology

**FIGURE 4:**
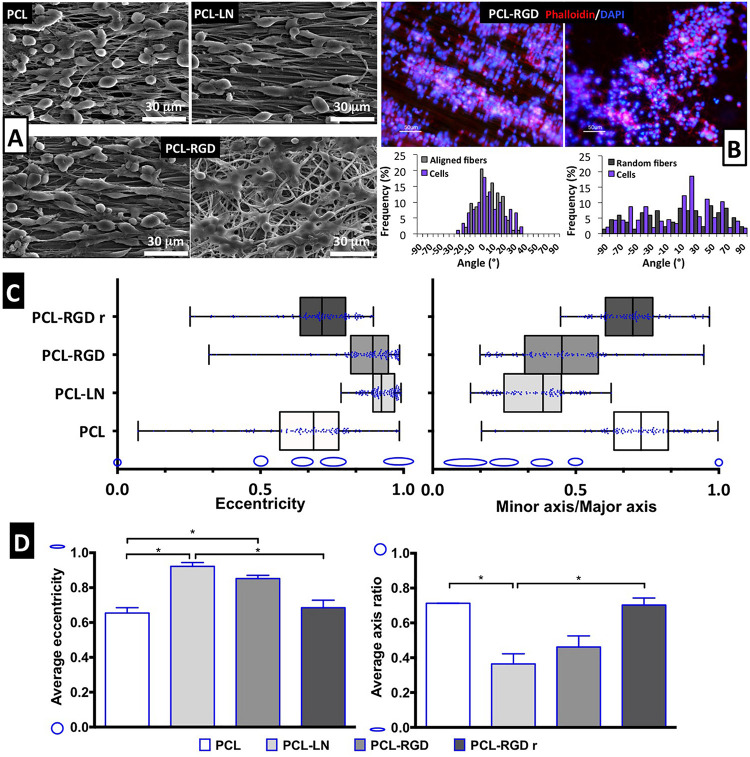
Evaluation of cell morphology and organization after CGR8-NS cell expansion on the PCL nanofibers. **(A)** SEM images after 3 days of expansion; scale bar 30 μm. **(B)** Image of cellular cytoskeleton staining with rhodamine phalloidin in PCL-RGD and PCL-RGDr, with respective histograms of cell alignment versus fiber arrangement; scale bar 50 μm. **(C)** Box-and-whisker plot for eccentricity (*E* = 0, perfect circle; *E* = 1, elongated shape) and aspect ratio (AR = 1, perfect circle; AR = 0, elongated shape) for cellular elongation quantification. The box boundaries represent the 25th and the 75th percentile; the straight line inside is the median. A minimum of 50 cells were measured. **(D)** Average values for eccentricity and aspect ratio. The shape corresponding to discrete eccentricity and axis ratio values is illustrated on the *y* axis. Error bars represent standard error of the mean; *p < 0.05 (*n* = 2).

Scanning electron microscopy images were obtained for each of the conditions to better characterize the morphology of the NSCs cultured on the different nanofibers (Figure 4A). It is possible to observe that NSCs are spread and round-shaped when cultivated on PCL-RGD random and pristine PCL–aligned nanofibers. In contrast, cells cultured on PCL-LN– and PCL-RGD–aligned nanofibers had an elongated shape, following the nanofiber axis orientation. Fluorescence microscopy images obtained with rhodamine phalloidin, which stains the F-actin fibers of the cellular cytoskeleton, on PCL-RGD and PCL-RGDr fibers (Figure 4B) also reveal an ordered distribution of the cells, aligned in the direction of the PCL-RGD nanofibers and a more dispersed and spread cellular distribution in the random nanofibers. The quantification of these differences is represented in Figure 4B, with histograms showing the measured angles of the stained F-actin fibers with reference to the direction of the nanofibers. The cells in the aligned fibers are distributed across a narrower angle range of ±30°, while in the non-aligned nanofibers the angle distribution to which cells occupy is wider over the interval of ±90°.

Cell shape analysis results are shown in Figure 4C. The box-and-whisker distributions profiles are identical between both shape parameters, with both PCL-LN and PCL-RGD boxes located in values that correspond to an elongated cell shape. Tighter population distributions in terms of eccentricity are found for PCL-LN and aligned PCL-RGD. The estimated averages of both shape parameters (Figure 4D) are also in agreement with the previous observations and differ identically for each nanofiber condition. The average cell eccentricities in PCL-LN reached a value of 0.92 ± 0.03 (very close to 1), being significantly different to PCL and PCL-RGDr, with average eccentricities of 0.65 ± 0.04 and 0.72 ± 0.13, respectively. In PCL-RGD scaffolds, the NSCs show also higher elongation with average eccentricity of 0.85 ± 0.09, comparable to PCL-LN. Eccentricity of PCL-NH2 fibers was also estimated (Supplementary Figure 6) at a value of 0.62 ± 0.03 (and aspect ratio of 0.73 ± 0.03), values similar to the pristine PCL fibers. This result suggests that the adhesion peptide/protein biological motifs on the scaffolds establish important interactions with the cells.

### NSC Differentiation in the Nanofibers

**FIGURE 5:**
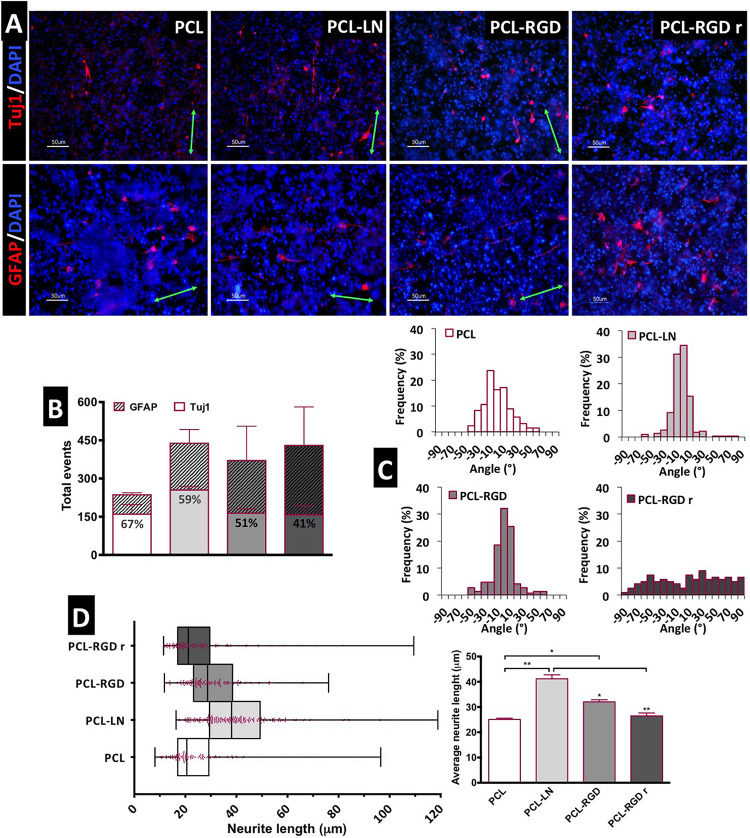
Evaluation of *in situ* CGR8-NS differentiation: **(A)** Immunofluorescence images of the expression of the neuronal marker βIII-tubulin (Tuj1) and astrocyte marker GFAP (green arrows indicate the fiber direction). **(B)** Total differentiated cells (events) counted. **(C)** Histograms of neuron alignment distribution. **(D)** Representation of the neurite length distribution (on the left). The box boundaries represent the 25th and the 75th percentile; the straight line inside the box is the median. On the right, average neurite lengths; a minimum of 50 cells were measured; *p < 0.05; **p < 0.01 (*n* = 2).

After 11 days of NSC expansion on the nanofiber scaffolds, neuronal differentiation was induced for 15 days. In Figure 5A, immunofluorescence images show the expression of Tuj1 and GFAP, indicating the presence of neurons and astrocytes, respectively, in all the nanofibers conditions.

The evaluation of the number of differentiated cells in each of the nanofibers was performed based on the immunofluorescence images, taken at day 15 of the differentiation protocol. The cells expressing Tuj1 or GFAP were counted and considered as “differentiated cells,” cells expressing neither of these two markers are most likely non-differentiated immature neural progenitors. The relative percentage of neurons and astrocytes for each condition was estimated (Figure 5B). Overall, the results suggest a higher number of differentiated cells on PCL-LN and PCL-RGD nanofibers and a lower number of differentiated cells in pristine PCL nanofibers (Figure 5B). Regarding the relative percentage of neurons/astrocytes, higher percentages of neurons (values indicated in the Tuj1 bars) were estimated in pristine PCL (67%) and PCL-LN (59%), similar percentages of neurons and astrocytes were estimated for aligned PCL-RGD (∼50%), and a higher percentage of astrocytes was found for PCL-RGDr (59%).

The number of differentiated cells (differentiation events) was normalized with the total number of cells, assessed by the number of DAPI-stained nuclei to give an approximate overview of the differentiated cells relative to the total number of cells in culture (Supplementary Figure 7). In general, a low percentage of differentiated cells relative to the total number of cells in culture was obtained (Supplementary Figure 7) ranging from the highest, obtained in PCL-LN (7% of neurons and 3% of astrocytes), to the lowest, in pristine PCL (3.4% of neurons and 1.4% of astrocytes), with the percentage of Tuj1 expressing cells higher for all the conditions.

The alignment histograms (Figure 5C) show the influence of the nanofiber organization on the differentiated cell orientation. In aligned pristine PCL matrices, cells were distributed along a ±50° orientation angle range, evidencing a well-defined peak of cell alignment. Regarding PCL-LN and PCL-RGD, sharper histogram peaks are obtained, indicating that a higher percentage of the cell population follows the fiber alignment, approximately within a narrower range of ±30° orientation angles, for both conditions. Cells cultured in PCL-RGDr present a wide distribution of alignment angles within the broad interval of ±90° orientation distribution, highly in contrast with the alignment peaks in the histograms determined for the aligned nanofiber conditions.

The neurite lengths profile distribution, displayed in the box-and-whisker plot, and the corresponding average neurite lengths are represented in Figure 5D, left and right, respectively. Tight length distribution (narrower boxes) was observed in PCL-RGDr and in non-functionalized PCL nanofibers, where shorter neurites developed (26.5 ± 1.4 and 25.1 ± 1.2 μm, respectively). A broader length distribution (wider box) was found in PCL-LN nanofibers with the longest average cellular extensions determined (41.07 ± 1 μm). The higher neurite lengths found in PCL-LN differ significantly from the remaining conditions, especially with pristine PCL and PCL-RGDr. The lowest neurite extensions found in pristine PCL also differed significantly from the PCL-RGD condition.

## Discussion

In the current study, four types of electrospun nanofibers were produced, and the NSC response and interaction with the scaffolds were assessed. Aligned pristine PCL scaffolds, i.e., aligned PCL nanofibers without any biological functionalization, were used to evaluate the effects of fiber alignment on the NSCs; aligned PCL fibers functionalized with LN (PCL-LN) and GRGDSP (PCL-RGD) were used to evaluate the synergistic effects of nanofiber alignment and the biological cell adhesion motifs; randomly oriented nanofibers functionalized with GRGDSP (PCL-RGDr), with a dispersion of diameters and fiber alignments, were also used to assess the effect of a fibrous disordered structure on NSCs. The effect of each of the four scaffolds in cell culture was analyzed according to (i) cell organization, through estimation of cell alignment after 11 days of expansion and at the end of the differentiation stage; (ii) cell morphology, estimated by cell eccentricity or axis ratio at the end of the expansion stage; and (iii) cell differentiation, determined at the end of differentiation by the relative percentage of Tuj1 (neurons) and GFAP (astrocytes)–positive cells, as well as the length of the neurites developed.

### The PCL Nanofiber Scaffolds

The distributions of diameters and relative orientation angles of the prepared aligned and random nanofibers are in agreement with other examples reported in literature (Wang et al., 2009, 2010; Cooper et al., 2011). For this particular study, heterogeneity in the random fiber meshes is desirable, as we were interested in producing a random matrix structure contrasting with the uniformity of the aligned nanofiber samples, to obtain two distinct types of morphologies with impact on NSC proliferation and differentiation (Yang et al., 2005; Nisbet et al., 2008; Christopherson et al., 2009; Horne et al., 2009; Hackett et al., 2010). The highest average diameter was obtained for random fibers when compared with aligned fibers, which can be attributed to the different collector material and configurations, because the other main conditions for the electrospinning process, polymer concentration/solution viscosity, applied potential, flow rate, and solvent (Teo and Ramakrishna, 2006; Nezarati et al., 2013) were kept the same.

The fiber mesh density is also an important feature as a parameter for cell culture. The use of scaffolds with similar fiber densities is important for the consistency of the cell culture experimental results. The fiber density should be high enough to permit cell–cell and cell–material contact but should also provide enough porosity to ensure good culture media infiltration. Highly compact meshes may perform, at the cell length scale (∼30 μm), as a membrane or film, with less pronounced three-dimensional (3D) structure (Stevens and George, 2005; Agarwal et al., 2008), and microscopic visualization and characterization of cultured cells may be difficult. However, the fiber density should facilitate the covering of the glass surface of the slide, avoiding cell adhesion to the glass, and inaccurate estimation of cell proliferation on the nanofibers. To avoid the effects mentioned above, a fiber density of 70–80% (i.e., a “surface porosity” of 20–30%) was selected for this study. This fiber density corresponds to a rank of 4.5 on the “five-scale fiber-density ranking” here established by collecting and testing a series of nanofibers with different mesh densities. The selected nanofiber samples, with a uniformly dense mesh, show evidence of a porous structure. While pore size and volumetric porosity were not estimated, fiber density (and “surface porosity”) provides a semiquantitative metric for scaffold porosity. Therefore, the two types of scaffolds selected for this study provide high surface areas and similar fiber densities, but very different morphologies, in terms of fiber diameter and alignment, allowing us to compare cell responses (e.g., organization and shape) to these geometries.

The next step for the preparation of the nanofibers for cell culture was to covalently bind LN and the GRGDSP peptide on the material surface. Covalent attachment of biological motifs has been shown to be advantageous for tissue engineering applications, especially for long-term cell culture, providing a more stable layer of proteins on the culture surface, in comparison with physical adsorption (Yoo et al., 2009; Ghasemi-Mobarakeh et al., 2010; Zander et al., 2010). Therefore, a protocol for covalent immobilization of LN and GRGDSP onto the PCL was applied in this study. The biological factors were immobilized successfully, with higher efficiency of immobilization for the GRGDSP peptide (83.8 ± 16.8%) when compared with LN (22.1 ± 1.5%). Considering the amine content for LN and GRGDSP and orthogonal even distribution of these species at the fiber scaffold surface, estimated equivalent amine immobilization densities would translate on molecules densities of 7.2 nmol cm^–2^ and 0.60 pmol cm^–2^ for GRGDSP and LN, respectively. Such molecular density would imply that LN molecules would be at 18.2 nm from each other (assuming LN size and shape as a cross-like structure with 8 nm for 70- to 90-nm size). Regardless of the potential inaccuracy of these estimations, they provide an order of magnitude estimation of peptide and protein coverage of the fiber surface.

In fact, the large 810-kDa multidomain glycoprotein of LN and the small 587-Da synthetic linear peptide GRGDSP differ greatly in size and structure (Beck et al., 1990; Hersel et al., 2003). The size of GRGDSP might be advantageous in terms of reactivity (easy diffusion and chemical lability), making a more effective use of the NH_2_ groups available at the PCL-NH_2_ fiber network and resulting in higher functionalization efficiency. It is important to note that during the glutaraldehyde reaction step, in addition to the crosslink between biomolecule and amine groups in the PCL-NH_2_ fibers, interbiomolecule crosslinking can occur, implying several layers of linked biomolecules. Overall, it can be said that it was possible to successfully immobilize LN and RGD in the nanofibers, providing biological motives for cell adhesion in the functionalized nanofibers.

### NSC Culture on the PCL Nanofibers

The nanofiber scaffolds prepared were demonstrated to be appropriate substrates for the proliferation of CGR8-NS cells. The cells retained expression of NSC markers, as well as the differentiation potential, and in the PCL-LN and PCL-RGD nanofibers improved cell adhesion; higher growth rates and final cell numbers were observed, in relation to pristine PCL. Studies report variable tendencies in cell proliferation in aligned and random matrices. Increased proliferation of neural progenitor cells (NPs) was found in aligned collagen nanofibers (Wang et al., 2011), of Schwann cells in PCL-PLGA (Subramanian et al., 2012), of human NPs in PCL nanofibers (Mahairaki et al., 2011), and mouse NSCs in PCL/collagen nanofibers (Hackett et al., 2010). Others report no statistically significant differences between aligned and random matrices as with PC-12 cells in PCL functionalized with LN and collagen (Zander et al., 2010), with cortical NSCs in PCL-BDNF nanofibers (Horne et al., 2009) or with Schwann cells in PCL–chitosan nanofibers (Cooper et al., 2011). An enhanced cellular expansion is associated with a geometry and biochemistry of the substrate that offers more accessible contact points for cells to adhere and a degree of porosity that facilitates increased diffusion, better biochemical enrichment of the substrate, and cell infiltration (He et al., 2010; Wang et al., 2011; Wheeldon et al., 2011). The highest final cell numbers were obtained with the PCL-RGD and PCL-RGDr. The random nanofiber structure (PCL-RGDr) permits the cells to efficiently adhere and to proliferate, even if the cells are organized in several directions, without the unidirectional structure observed in aligned nanofibers. Comparable levels of cell expansion were observed with PCL-LN. Although LN has the potential to provide more specific interaction with NSC integrins, the unpredictable orientation of the biomolecule after immobilization in the fibers as well as the interactions with the substrate (water affinity, morphology, charge) might restrict the access of the cellular integrin receptors to LN (Hersel et al., 2003; Nakaji-Hirabayashi et al., 2012). Moreover, protein crosslinking by glutaraldehyde may lead to some extent of protein denaturation, which can also impair cell adhesion and proliferation (Migneault et al., 2004). The surface of the pristine PCL nanofibers facilitated cellular attachment, possibly mediated by adsorption of proteins present in the culture media, permitting NSCs to proliferate but to a lower extent. PCL has a relatively high hydrophobic character, described by high contact angles (θ > 90°) on solid surfaces (Fang et al., 2011; Yuan and Lee, 2013), and therefore, cell adhesion is more difficult to occur. Nevertheless, cellular survival and proliferation in untreated PCL random nanofibers have been previously reported (Schnell et al., 2007; Chew et al., 2008; Nisbet et al., 2008).

### NSC Organization and Morphology

Stem cell morphology and differentiation potential are influenced by the physical substrate, and in particular, for highly polarized cells, such as NSCs, substrate morphology is often determinant (Ingber, 2003; Bettinger et al., 2009; Qi et al., 2013). The parameters here analyzed, cell alignment and shape (eccentricity and axis ratio), demonstrated the influence of nanofiber morphology on cell orientation and shape. As expected, NSCs were able to align and elongate, extensively exhibiting a highly bipolar morphology, especially in the presence of adhesion factors in PCL-LN and PCL-RGD. Aligned but less elongated cell populations were found in pristine PCL fibers, and cells with a round and spread shape morphology were found in PCL-RGDr. Note that it was reported that contact angle dropped from about 80° in pristine PCL films to the range 59–68°, both for PCL films aminolysed or further functionalized with proteins (Zhu et al., 2002). In the current study, similar NSC eccentricity and aspect ratio were obtained for cultures on pristine or aminolysed PCL fibers, suggesting that the adhesion peptide/protein biological motifs on the scaffolds establish important interactions with the cells.

Similar illustrative examples regarding the effect of the morphology were also described with adult NSCs in LN-coated PCL nanofibers (Lim et al., 2010) and with NPs after 3 days of culture in PCL poly-ornithine/LN–coated nanofibers, where cells in the random substrate show a spread and less polar morphology (Mahairaki et al., 2011).

### NSC *in situ* Differentiation and Cell Morphology

The NSCs used in this work were able to differentiate into neurons and astrocytes, in all nanofiber conditions tested. However, a relatively low percentage of differentiated cells was obtained, taking into account an average of 10–40% differentiated neurons reported in the literature (Pollard et al., 2006a). The differentiation protocol used was optimized for culture in tissue culture plates, and the conditions described may require further optimization when adapted to culture on nanofiber scaffolds. In this study, the differentiation stage was initiated from cell cultures at very high density, over a relatively short period (2 weeks compared to 5–6 weeks in other studies) and without the use of specific factors to direct differentiation or support survival of differentiated cells. Therefore, further improvement on differentiation efficiency may be attained by optimization of initial cell density before inducing differentiation, by adjusting the concentration of growth factors and/or the length of the differentiation steps, permitting a longer differentiation time and by supplementing the culture with specific molecules, such as neurotrophic factors (e.g., BDNF), to enhance neuronal differentiation (Pollard et al., 2006a). Other strategies to enhance differentiation include the application of physical stimuli using electroconductive materials (Garrudo et al., 2019a,b) and/or applying electrical stimulation (Pires et al., 2015).

In relation to the total cells in culture, the relative percentage of neurons was higher under all the conditions. When considering the percentage of neurons in relation to the number of differentiated cells, neurons were in higher proportion in aligned PCL and PCL-LN, in even percentage with astrocytes in PCL-RGD, and in lower percentage in PCL-RGDr. This tendency suggests that the alignment of the fibers promotes neuronal differentiation, but RGD functionalization favored differentiation toward astrocytes. Still, it is difficult to draw strong conclusions, because the overall differences (Figure 5B) were found not to be statistically meaningful and were associated with a considerable error.

Previous studies reported that substrate dimensions and alignment has an influence on directing neuronal differentiation of adult NSCs (Lim et al., 2010) and alignment of neurites parallel to nanofiber direction (Silantyeva et al., 2018; Philip et al., 2019). It was suggested that aligned substrates favored the survival of neural progenitors in detriment with non-neuronal progenitors, and nanofiber dimensions influenced cell–substrate interactions (Lim et al., 2010). Other studies showed that neuronal lineage differentiation was favored in aligned nanofibers with negligible expression of astrocytes and oligodendrocytes markers (Mahairaki et al., 2011), and a greater extent of neuronal differentiation (80%) (Bakhru et al., 2011) or accelerated neural differentiation (Silantyeva et al., 2018) was observed when NSCs were cultivated on fiber substrates. In the current study, the apparent prevalence of astrocytes in the random nanofibers can be explained by the physical cues provided by the random distribution of fiber diameters and fiber alignments in the PCL-RGDr. Such morphology presents discrete scattered surface contact points for cell adhesion, that can promote broader spread morphology, and hence the astrocyte lineage can be favored under these conditions. In non-functionalized PCL nanofibers, although the number of differentiated cells was smaller, an increased number of neurons were observed. Based on previous reports, aligned fiber morphology is able to promote neuronal lineage differentiation, and the high percentage of neuronal cells within the detected differentiated cells could be explained by the effect of the alignment of the matrix itself.

The neuron alignment and neurite extension are in agreement with previous observations for NSCs, illustrating the effect of the functionalized material in cell organization and morphology (Yang et al., 2005; Zander et al., 2010; Wang J. et al., 2012; Smith Callahan et al., 2013). The higher cell adhesion to the nanofibers provided by the LN or GRGDSP motifs and the unidirectional organization of the substrate contributed to guide the cellular distribution and to a more extensive neuronal elongation. The shorter neurite length found in PCL-RGDr is likely due to the disordered disposition of the nanofibers, where the cells developed a more spread and multidirectional morphology. The neurite extension of Tuj1-positive neuronal cells was clearly improved by the nanofiber alignment and in the presence of adhesion factors, in particular LN.

## Conclusion

The nanofiber scaffolds prepared were found to be suitable for the proliferation and differentiation of mouse ESC-derived NSCs. This NSC population was able to proliferate in adherent monolayer and to maintain the multipotent potential. Under differentiation conditions, the morphology of the substrate was shown to have a role in cell fate. The results obtained show the existence of a synergistic effect of substrate morphology and specific cell adhesion motifs on NSC morphology, proliferation, and differentiation. The functionalization with biological motifs promoted cellular adhesion to the fibers, leading also to increased NSC proliferation. Non-functionalized aligned PCL nanofibers were able to promote cell alignment but performed poorly in promoting NSC elongation. In contrast, cellular elongation was improved in aligned scaffolds functionalized with GRGDSP or LN, which promote specific adhesion points according to the uniaxial matrix structure. Randomly distributed GRGDSP functionalized fibers also improve cell adhesion, but because of the lack of a single direction axis, the cellular orientation follows a distribution in arbitrary directions. These observations are valid for undifferentiated NSCs after 11 days of proliferation and for cells after the differentiation step.

The nanofiber morphology directed neuronal lineage and neurite elongation in the aligned matrices especially in the presence of the adhesion motifs. PCL-LN fibers led to the highest percentage of neurons within the differentiated cells, as well as to the longer average neurite length. In contrast, random GRGDSP matrices were found to be relatively preferential for astrocyte maturation. PCL-GRGDSP–aligned substrate was found to promote cellular adhesion, elongation, and proliferation, also permitting neuronal lineage differentiation, although to a lower extent than PCL-LN.

The prepared scaffolds, in particular when LN is used, can be considered promising for NSC culture and may, in the future, be applied in therapies for regeneration of CNS injuries. Moreover, cellular alignment is an interesting property that can be beneficial to tissues requiring such geometry, such as in spinal cord injury repair. One of the major problems of biomaterial scaffolds is the ability to maintain cellular viability when applied *in vivo*. However, as *in vivo* studies were out of the scope of the current study, additional improvements and scaffold development may need to be explored. Depending on the type of CNS tissue (spinal cord or brain), a deep assessment of the *in vivo* conditions is important in order to design and develop the most compatible material for *in situ* cellular delivery complemented with trophic factors and/or drugs to provide an environment suitable for cellular survival and function. Blends of polymers to optimize the biomaterial stiffness, combination of the fibers with a hydrogel to gradually release of biochemical cues or assemblies of microstructures and nanostructures to provide specific architecture and 3D environment, are interesting concepts for future scaffold design.

## Data Availability Statement

The raw data supporting the conclusions of this article will be made available by the authors, without undue reservation.

## Author Contributions

FF, MA, and CR contributed to original idea, experimental plan, and manuscript writing. MA, CR, and IF contributed to preliminary results. MA and IF assisted by CR and FF contributed to electrospun and cell work experiments. MA with guidance by CR and FF contributed to data analysis and figures preparation. CR, MD, RL, JC, and FF contributed to scientific guidance and discussions, laboratory space, and funding. All authors revised the manuscript.

## Conflict of Interest

The authors declare that the research was conducted in the absence of any commercial or financial relationships that could be construed as a potential conflict of interest.
