# Supplementary material for: Functionalization of Electrospun Nanofibers and Fiber Alignment Enhance Neural Stem Cell Proliferation and Neuronal Differentiation
Source: Front Bioeng Biotechnol. 2020 Oct 26;8:580135. doi: 10.3389/fbioe.2020.580135 (PMC7649414; doi:10.3389/fbioe.2020.580135)
Supplement: Supplementary file 1 [file Data_Sheet_1.docx]

### Supplementary Information

**Title**

Functionalization of electrospun nanofibers and fiber alignment enhance neural stem cell proliferation and neuronal differentiation

**Authors**

Miriam C. Amores de Sousa^1^, Carlos A. V. Rodrigues^1^, Inês A. F. Ferreira^1^, Maria Margarida Diogo^1^, Robert J. Linhardt^2^, Joaquim M. S. Cabral^1^, Frederico Castelo Ferreira^1^*

^1^Institute for Bioengineering and Biosciences, Department of Bioengineering, Instituto Superior Técnico, Universidade de Lisboa, Av. Rovisco Pais, 1049-001 Lisboa, Portugal

^2^Center for Biotechnology & Interdisciplinary Studies, Department of Chemistry & Chemical Biology, Rensselaer Polytechnic Institute, Biotechnology Center 4005, Troy, NY, 12180, USA.

*E-mail: frederico.ferreira@tecnico.ulisboa.pt

Figure S1 –PCL nanofiber functionalization with LN and GRGDSP: (a) SEM images of PCL nanofibers before and after aminolysis treatment (scale bar: 5 (left) and 7 (right) μm); (b) Representation of the suggested surface functionalization reaction steps for possible functionalization of PCL material through covalent bonding; (c) Quantification of equivalent NH_2_ groups in nanofibers (inset shows in detail PCL-NH_2_ vs. PCL-LN) and initial solutions of LN and RGD peptide(“LN initial” and “RGD initial”, respectively). (d) Reference calibration curve of ninhydrin–NH_2_ absorbance (538 nm) as a function of graded concentrations of HDA in 1:1 v/v of 1,4-dioxane/isopropanol solutions. Error bars represent SD.


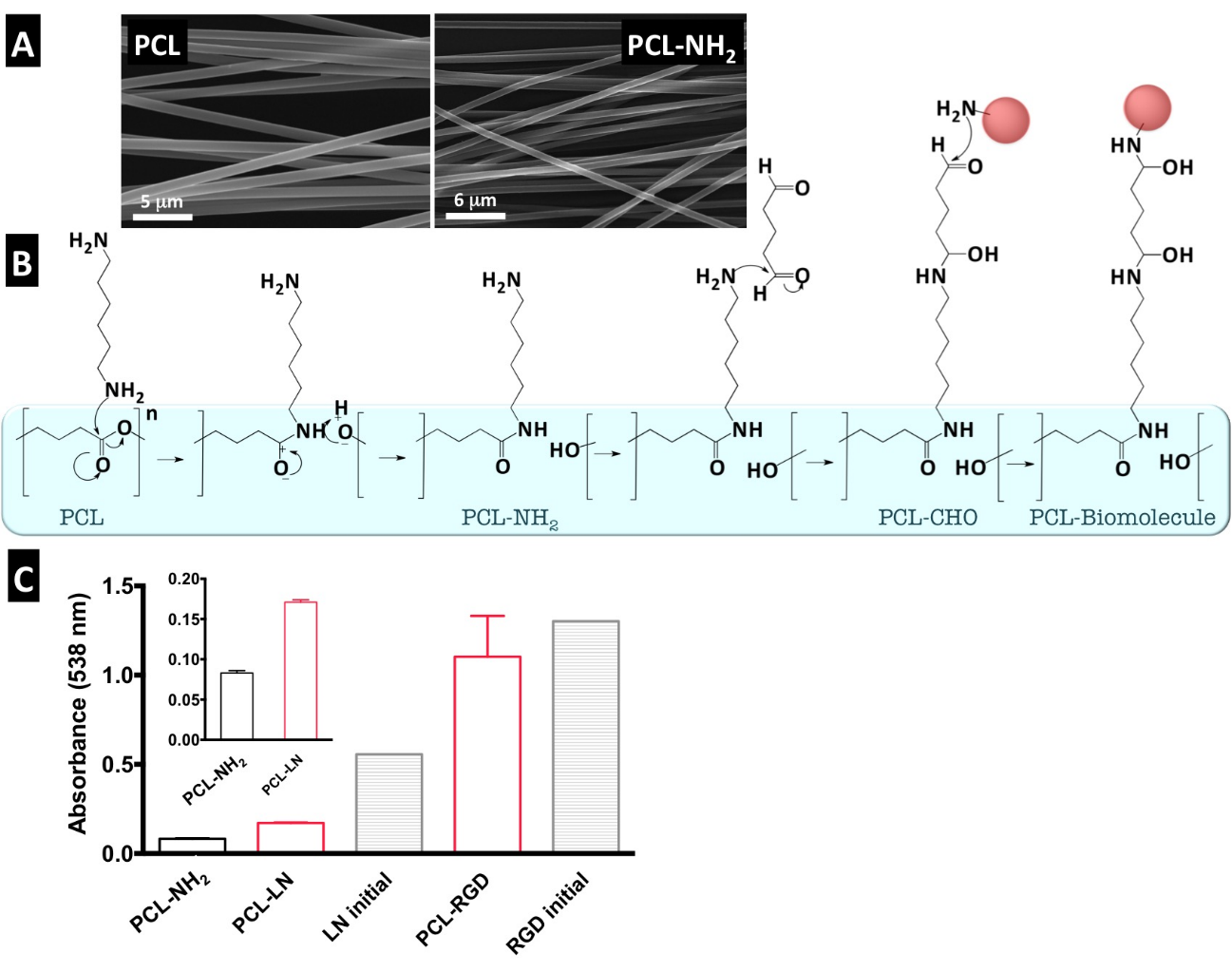

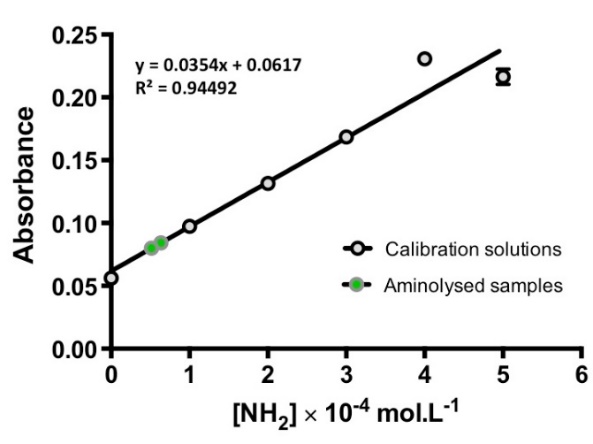


**D**


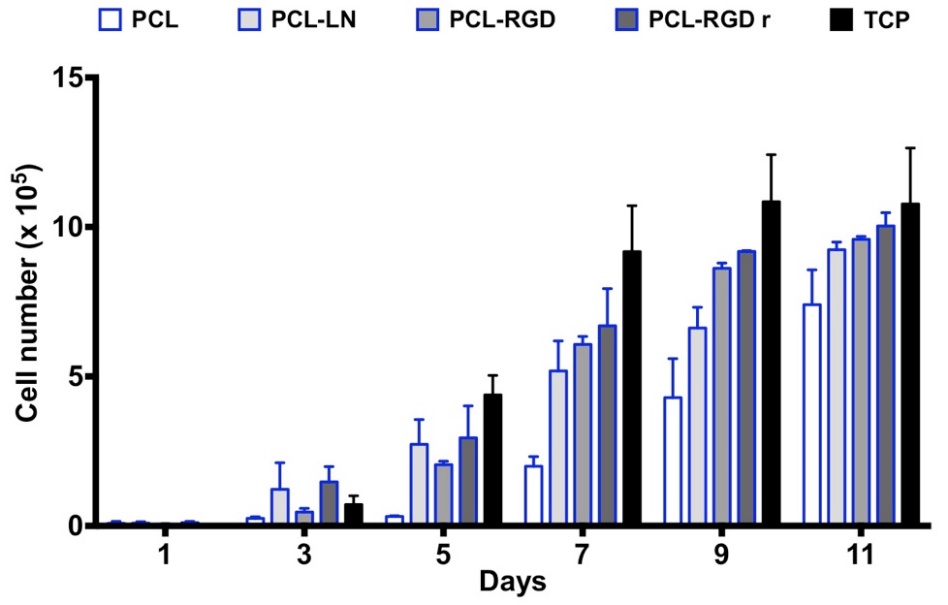


Figure S2 – Cell proliferation profile of CGR8-NS cells in PCL nanofiber scaffolds compared with the cellular growth in a 24 well polystyrene tissue culture plate (TCP).


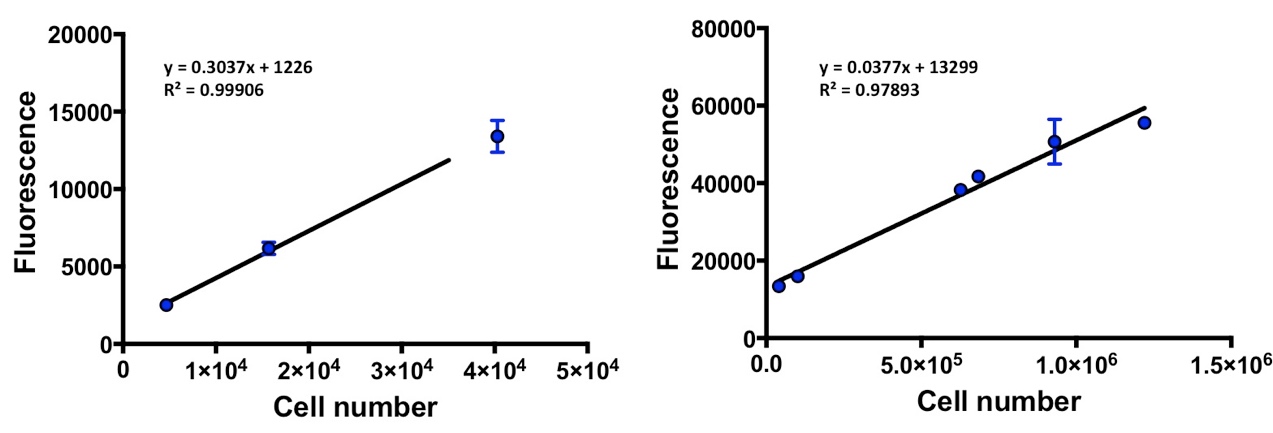


Figure S3 – Calibration lines for the fluorescence of the Alamar Blue reduced product with the CGR8-NS cell number on nanofiber scaffolds. (n=2); Plot for cell numbers from 5000 - 40000 and from 40000 - 1000000.


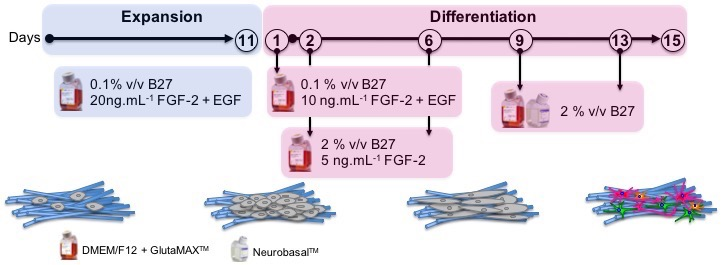


**Figure S4 – Differentiation protocol applied to the NSCs after 11 days of expansion on the nanofiber scaffolds.**


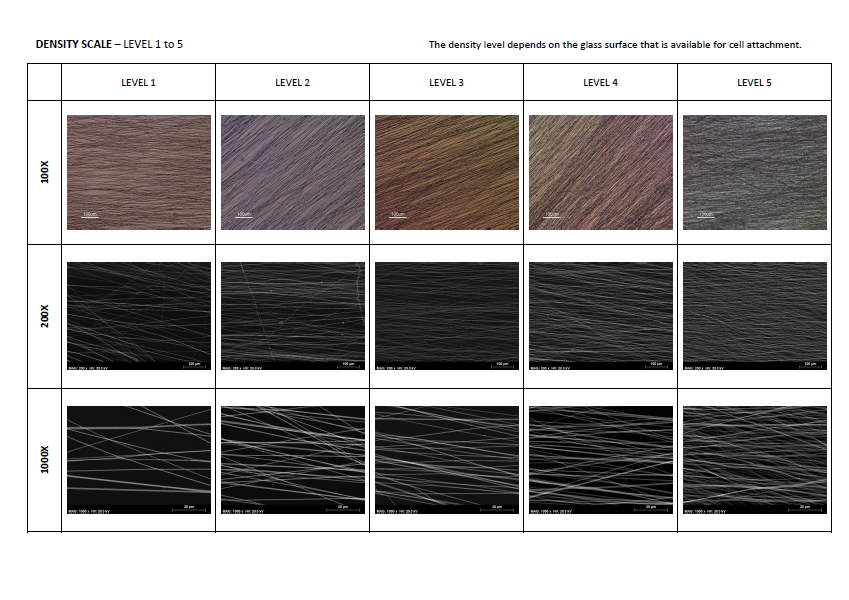


Figure S5 – SEM and optical microscope images illustrative of the nanofiber density levels considered. Scale bars are 100 μm for optical microscope images with magnifications of 100X and 200X, and 30 μm for images with magnifications of 100X.


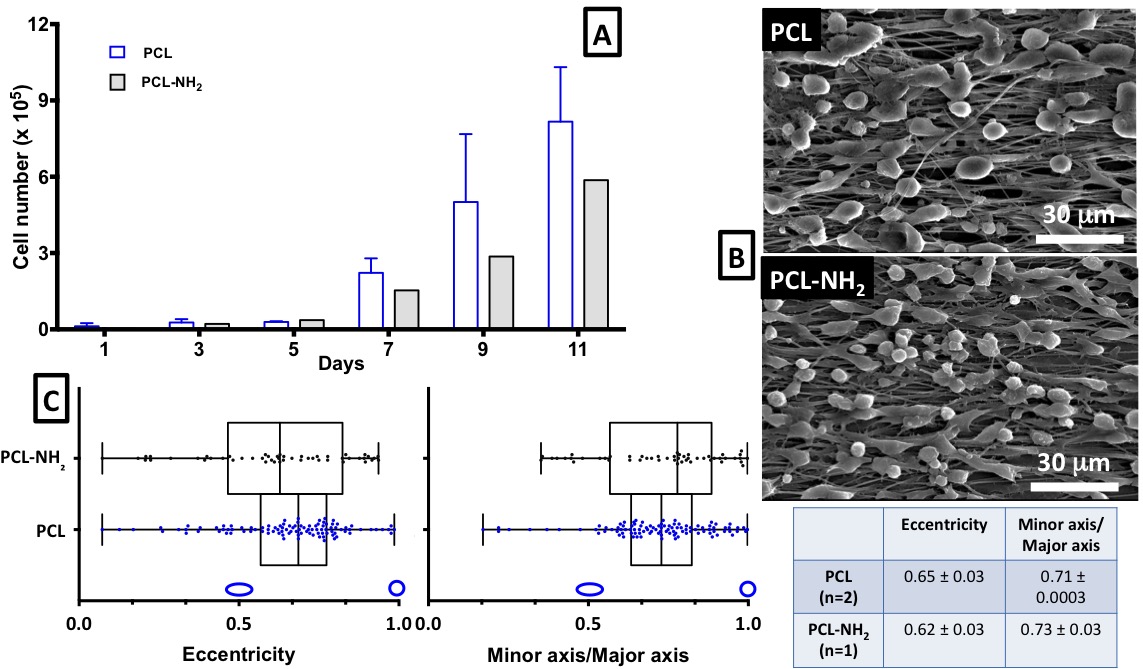

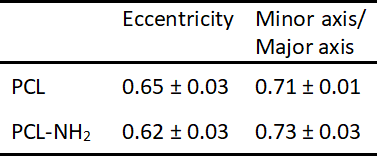


Figure S6 – Comparison of the cell proliferation and morphology in the PCL pristine (PCL) and aminolysed PCL nanofibers (PCL-NH2): (a) evolution of the cell growth over time in PCL-NH2 (the represented bars correspond to one replica n=1) compared with PCL (n=2); (b) SEM images after 3 days of culture; (c) box and whiskers plot representation of the quantification of the cell morphology parameters, eccentricity and AR (minor axis/major axis).


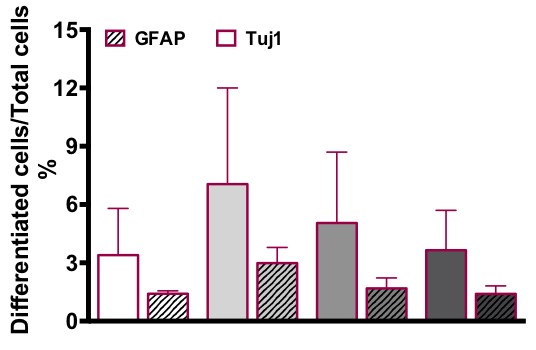


Figure S7 - Estimated percentage of differentiated neurons and astrocytes relative to the total cells in culture; (n=2);
